# Supplementary material for: Adherence to different complementary feeding methods in the first year of life: A randomized clinical trial
Source: PLoS One. 2023 Nov 2;18(11):e0289696. doi: 10.1371/journal.pone.0289696 (PMC10621984; doi:10.1371/journal.pone.0289696)
Supplement: Attachment — Submitted filename: Response Letter PLOS ONE 230623.docx [file pone.0289696.s001.docx]

Manuscript title: Adherence to different complementary feeding methods in the first year of life: a randomized clinical trial

Manuscript ID: PONE-D-22-20911

Journal: Plos One

Article type: Original Research

Editor-in-Chief of Plos One,

The authors are grateful for the appreciation and the opportunity to publish the manuscript entitled **"Adherence to different complementary feeding methods in the first year of life: a randomized clinical trial"**. The authors also appreciate the time spent by the reviewers with important collaborations for the qualification of scientific production. Below are the responses to the comments.

All changes in the manuscript are highlighted in yellow. A detailed point-to-point response follows for each reviewer is described below.

The authors are available for questions or comments about the article.

Best regards,

The authors,

Universidade Federal do Rio Grande do Sul (UFRGS)

2th version:

We note that your manuscript is not formatted using one of PLOS ONE’s accepted file types. Please reattach your manuscript as one of the following file types: .doc, .docx, .rtf, or .tex (accompanied by a .pdf).

If your submission was prepared in LaTex, please submit your manuscript file in PDF format and attach your .tex file as “other.”

**Answer: The authors submitted the manuscript file in PDF format and attached as file.**

Please update your submission to use the PLOS LaTeX template (<http://journals.plos.org/plosone/s/file?id=SyJ3/plos-latex-template.zip>). We cannot proceed with your submission until your manuscript has been converted to this format.

**Answer: The authors adjusted the submission to use the PLOS LaTeX Template.**

Thank you for stating:

“No - some restrictions will apply;
The datasets generated and/or analyzed during the current study are not publicly available because they are part of a larger study, with analyzes still being carried out. For this reason, the authors of this article have not released the data for public access on any online platform. If requested, the corresponding author can send them directly by email."

Please provide an updated data availability statement with the following information:

A) Please clarify if the data are ethically or legally restricted. If so, please state the reason(s) why the data are restricted.

**Answer: The datasets generated and/or analyzed during the current study are not publicly available due to longitudinal data collection being ongoing but are available from the corresponding author upon reasonable request.**

B) If the data are restricted by an ethics committee or an Institutional Review Board (IRB), then please state the name of the IRB or ethics committee imposing these restrictions.

**Asnwer: The data are not restricted by an ethics committee.**

C) Please provide the contact information to which requests for data may be sent. Preferably, this would be an institutional email address to your IRB or ethics committee. If you are unable to provide a non-author contact point capable of handling data access requests, please explain why.

**1th version:**

Please use the space provided to explain your answers to the questions above. You may also include additional comments for the author, including concerns about dual publication, research ethics, or publication ethics. (Please upload your review as an attachment if it exceeds 20,000 characters).

1. Please ensure that your manuscript meets PLOS ONE's style requirements, including those for file naming. The PLOS ONE style templates can be found at

<https://journals.plos.org/plosone/s/file?id=wjVg/PLOSOne_formatting_sample_main_body.pdf> and <https://journals.plos.org/plosone/s/file?id=ba62/PLOSOne_formatting_sample_title_authors_affiliations.pdf>

**Answer: The authors adjusted the manuscript to PLOS ONE's style requirements. Besides that, the English idiom was revised.**

2. Please provide additional details regarding participant consent. In the ethics statement in the Methods and online submission information, please ensure that you have specified what type you obtained (for instance, written or verbal, and if verbal, how it was documented and witnessed). If your study included minors, state whether you obtained consent from parents or guardians. If the need for consent was waived by the ethics committee, please include this information.

**Answer: The information was inserted in the topic “ethical aspects” section.**

All responsible parties first signed the informed consent form online and, after that, they signed it in person in two copies, one for the research team and one for the child's guardian.

3. We note that the grant information you provided in the ‘Funding Information’ and ‘Financial Disclosure’ sections do not match. When you resubmit, please ensure that you provide the correct grant numbers for the awards you received for your study in the ‘Funding Information’ section.

**Answer: The authors appreciate your suggestion, and included the correct grant numbers in the “Funding Information” section.**

This study was supported by the Research and Events Incentive Fund–Hospital de Clínicas de Porto Alegre under grant number 2019-0230. Also, this study was financed by the Coordenação de Aperfeiçoamento de Pessoal de Nível Superior, Brazil (CAPES), and Conselho Nacional de Desenvolvimento Científico e Tecnológico (CNPq).

4. Please update your submission to use the PLOS LaTeX template. The template and more information on our requirements for LaTeX submissions can be found at <http://journals.plos.org/plosone/s/latex.>

**Answer: The authors adjusted the submission to use the PLOS LaTeX Template.**

Reviewer #1: This study aimed to assess adherence to three methods of food introduction: PLW, BLISS, or mixed (PLW and BLISS) at seven, nine, and 12 months of age. The topic is of interest as the adherence to different methods of weaning have not been investigated yet. My comments, as following:

Introduction:

- In addition to the Brazilian recommendations, also the WHO recommendations for complementary feeding should be cited.

**Answer: As suggested, the WHO recommendations were added.**

Materials and methods

“Each group was constituted of about eight pairs”: in the Results section, much more pairs are described. Please check this data.

**Answer: The information was removed and the phrases were rewritten.**

“The intervention sessions were conducted in groups of up to eight pairs of mothers and/or caregivers and children, according to the CF randomized method (PLW, BLISS or mixed).”

Intervention (5.5 months of age):

In the paragraph “Regarding PLW, …. without mixing and/or sieving the food. Food preparations should be separated so that children assimilate the flavors and characteristics of each one.” I was quite puzzled to read this statement; one of the main characteristics of the traditional purèed infant foods is to be composed of several food items mixed together (e.g. cereal creme + homogenized meat + vegetables). Why did authors choose to propose foods separately? Could they explain this choice?

**Answer: The authors appreciate your commentary. The Brazilian Ministry of Health advises that food consistency should be initially mashed without mixing and/or sieving the food. It also recommends that food preparations be separated on the plate so that children assimilate the flavors and characteristics of each one, stimulating children's learning. It is a Brazilian recommendation to not mix them.**

Data collection

“The interview was carried out by a researcher blinded to the method with a food frequency questionnaire and a food record prepared for this study”: How was the food record performed? Please add some details.

**Answer: The section has been rewritten providing more detail.**

“Follow-up data on family adherence to the CF method were collected until the end of the first year of life when children were seven, nine, and 12 months old.

The interview was conducted by a researcher blinded to the method using a standardized questionnaire designed for this study on food consistency and form of offering. It was questioned whether most of the time the food was offered liquefied, sieved, scraped, mashed with a fork, cut into sticks, or in the family consistency. Answers were provided as yes or no.

At the 7-month interview, by phone, at nine months of age, home visits, and 12 months of age, at the HCPA Clinical Research Center (CRC), Porto Alegre.

During the COVID-19 pandemic, at 9 months 50.7% (n=67) of the sample answered the questionnaires online, and at 12 months 80.3% (n=94) answered the online version of the same questionnaires.”

Results:

Results show that a substantial percentage of the children migrated from PLW/BLISS to mixed method (up to almost 100% both for PLW and BLISS with regard to consistency). However, infants were followed from the seventh until the 12th month of life. This is a crucial stage in the infant's neuromotor development, and it is therefore reasonable to assume that during these months the feeding pattern may change. Could the authors add a comment on this in the discussion?

**Answer: The authors appreciate your concern. In this paper, the authors focused mostly on the parents/guardians’ decision to practice or change the method of complementary feeding. Indeed, after the first year of life, it is expected that the child receives food in the same consistency as the family. A sentence was written in the discussion to clarify the theme.**

It is important to highlight that the present study focused mainly on the parents’ decision to practice or change the method of complementary feeding.

Discussion

- In the discussion authors say "mother-child pairs either adhered to methods by feeding mode or food consistency, so we chose to separate them." However, one of the key differences between a traditional PLW and BLISS is indeed the consistency of the foods offered. If authors aim to maintain a separate analysis for the feeding mode and consistency, I believe the reason for this distinction should be better clarified.

**Answer: The authors appreciate your commentary. The adherence to the methods was separated to analyze in detail the factors that led parents to the change in the method (if it was because of the mode of the offer, the consistency of food, or both). Although, just when the mode and the consistency were adequate for the method, it was considered that the family was following the method. A paragraph was written in the “*Adherence to methods of food introduction”* section to provide more detail.**

Adherence to the method was separated by feeding mode (if children received food from a spoon or took it to their mouths with their hand) and food consistency (if the food was liquidized, scraped, mashed, sieved, or cut into strips and the same consistency of the family’s food). Adherence to PLW was represented by parents who offered food with a spoon (feeding mode) and of a liquidized, scraped, mashed, or sieved consistency (food consistency) most of the time. Adherence to BLISS was represented by children who took food (feeding mode) of the same consistency as their family’s or in strips to their mouths (food consistency), and adherence to the mixed method was represented by those who used alternating feeding modes and consistencies most of the time.

- In the paragraph “Child-guided method …. The mothers’ will to follow CF recommendations provided by health professionals was another limiting factor ...”: in addition, more anxious mothers may more likely to choose a traditional weaning approach such as PLW, where there is more existing data and support from healthcare professionals. This could be another important influencing factor, please add a comment in regard. In regard, authors may consider the systematic review by D'Auria E, et al. Baby-led weaning: what a systematic review of the literature adds on. Ital J Pediatr. 2018

**Answer: The authors appreciate your commentary. The paragraph has been changed to:**

“Child-guided methods are associated with a low-control maternal feeding style, in which mothers control the children’s food intake less than mothers who use PLW; thus, parenting style could be an important factor to influence adherence to CF methods. The mothers' willingness to follow the recommendations of CF provided by healthcare professionals may limit adherence to the methods, as mothers report doing what was best for their children and not following a healthcare professional's guidance. Additionally, mothers who fed their children using the BLW method reported lower anxiety, lower scores of obsessive-compulsive disorder, lesser food restrictions, and greater awareness than mothers who opted for the PLW/traditional method. However, these results are from observational studies and require further investigation.”

Minor:

“an ANOVA test with Bonferroni post hoc test was used and.”: please complete the sentence.

**Answer: Thanks for your suggestion. The sentence was removed from the abstract because it refers to continuous variables that are only presented in the text and not in the abstract.**

Introduction: [14,11,14]: ref. 14 does not need to be repeated and some minor typos need to be checked

**Answer: Thanks for your suggestion. The text was corrected, as suggested.**

Reviewer #2: Summary of the research

This study aimed to assess adherence to three methods of food introduction; parent-led, baby led, and mixed, with follow-up at 7, 9 and 12 months. The intervention consisted of a one-hour group meeting with nutritionist who cooked examples of dietary foods and explained standardized information about the feeding method they had been randomised to. The study is described as a ‘randomised clinical trial’. Randomisation does indeed occur, specifically in to 3 treatment groups; parent led, baby led and mixed. However, there is no control group (e,g., standard care, treatment as usual, no intervention).

**Answer: Thanks for your suggestion. As written, the study was composed of three treatment groups: PLW group, BLISS group, and mixed group. The PLW group is the method usually recommended by health professionals to all children treated within the Brazilian unified health system, which includes public and private services. For this outcome, adherence, there was no need for a control group, as the study did not explore the consequences of the health outcomes, so there is no need for a baseline for determining the effectiveness of the study treatment.**

Results suggest that mixed method of feeding results in higher adherence and most parents from the other two groups also migrated to this method. The authors conclude this shows the feasibility of a mixed approach to guide families in the introduction of complementary feeding. The discussion considers some but not all of the limitations of the study. The research explores an important area, supplements previous work in the field and is novel in its exploration within a Brazilian sample. However, issues with the design, measurement and interpretation limit its value. There are some issues with details missing and clarity in expression.

**Answer: The authors appreciate your concern and modified the manuscript to improve your quality with the design, measurement, and interpretation limit its value.**

My overall recommendation would be major revisions.

Examples and evidence:

1. The study is described as a ‘randomised clinical trial’ and refers to parent-led weaning as a control. There is no control group (e.g., treatment as usual, standard care, no intervention); there are 3 treatment groups (parent-led, child led and mixed) and no control. It could be argued that there is no need for a control group within this trial as the study explores ‘adherence’ and not outcomes, so there is no need for a baseline for determining the effectiveness of the study treatment. However, authors should think carefully about the design and methodology of their trial, describe it accurately and explain the rationale behind it. They should not make claims that is a RCT with control group, if it is not.

**Answer: Thanks for your suggestion. As written, the study was composed of three treatment groups: PLW group, BLISS group, and mixed. The PLW group is the method usually recommended by health professionals to all children treated within the Brazilian unified health system, which includes public and private services. For this outcome, adherence to methods of food introduction, there was no need for adherence to methods of the food introduction group, as the study did not explore the consequences of the health outcomes, so there is no need for a baseline for determining the effectiveness of the study treatment.**

2. There are significant issues within the way adherence is measured, analysed and reported. For example on Page 6 the authors state that researchers ‘made a phone call and asked some questions, regarding food consistency and if children took food to mouth or received from mouth and another researcher analysed them’. What questions were they asked? How were responses interpreted, scored and analysed? At this stage it is unclear if responses were qualitative or quantitative, and if numerical responses were categorical (in which case what were they categories?) or on a continuum (again from what and to what?). In the discussion it becomes apparent that for feeding mode this was categorical with 3 groups (1. children took all or most of the food to their mouths; 2. children were fed by their parents; 3. they were equally fed by their parents and took food to their mouths). This needs to be much clearer within the methodology and also stated for feeding consistency. On page 8, Adherence to BLISS for consistency is described as ‘children who took food of the same consistency as their family’s or in strips to their mouths’ but it is not clear if this is all of the time, ‘all or most’ or a certain %. It is unclear if consistency adherence was also into 3 categories and if so, what those categories were.

**Answer: The “adherence to methods of food introduction” section has been rewritten and more information has been added, to clarify the information in manuscript.**

“Follow-up data on family adherence to the CF method were collected until the end of the first year of life when children were seven, nine, and 12 months old.

The interview was conducted by a researcher blinded to the method using a standardized questionnaire designed for this study on food consistency and form of offering. It was questioned whether most of the time the food was liquidized, scraped, mashed, sieved, or cut into strips and of the same consistency as the family’s food. Regarding the form of offer, those responsible answered whether the children took the food to their mouths alone or if they received complementary foods by spoon-feeding them up. Answers were provided as yes or no.

At the 7-month interview, by phone, at nine months of age, home visits, and 12 months of age, at the HCPA Clinical Research Center (CRC), Porto Alegre.

During the COVID-19 pandemic, at 9 months 50.7% (*n*=67) of the sample answered the questionnaires online, and at 12 months 80.3% (*n*=94) answered the online version of the same questionnaires.

Adherence to the method was separated by feeding mode (if children received food from a spoon or took it to their mouths with their hand) and food consistency (if the food was liquidized, scraped, mashed, sieved, or cut into strips and the same consistency of the family’s food). Adherence to PLW was represented by parents who offered food with a spoon (feeding mode) and of a liquidized, scraped, mashed, or sieved consistency (food consistency) most of the time. Adherence to BLISS was represented by children who took food (feeding mode) of the same consistency as their family’s or in strips to their mouths (food consistency), and adherence to the mixed method was represented by those who used alternating feeding modes and consistencies most of the time.”

This also brings to question the validity and quality of the measures used for adherence. A strength to this study is that it explores mode and consistency separately, however measuring adherence categorically, with poorly defined and reported categories, is a strong methodological flaw that really limits interpretation of the findings and the conclusions that can be drawn. What is meant by ‘all or most’? Previous studies (as noted in the discussion) define or measure this more tangibly/objectively. For example, Erickson et al., (2018) who assessed adherence to BLISS by calculating the percentage of daily food intake by weight (g) consumed by children who fed themselves/were fed by an adult. Komninou et al., (2019) classified adherence to strict BLW as children feeding themselves 90% of the time or more.

**Answer: The authors appreciate your concern. “Most of the time” was defined as the child's usual diet or 75% of meals. Furthermore, considerations about the limitation in the way of assessing adherence were added to the discussion section.**

“This research has some limitations. Just under 30% of the mothers who met the inclusion criteria did not attend the intervention; these mothers had lower schooling and family income than those included. In Brazil, maternity leave lasts four months in most companies and our intervention occurred at 5.5 months of the children's lives, therefore, there may be mothers unable to participate in this study. Family income and schooling level in our sample were higher than in the general population, which compromises the generalizability of results. The sample was spontaneously recruited mainly from the target social networks, which may result in mothers previously interested in complementary feeding. The sample size may have been insufficient to obtain significant results in the tests performed to assess the association. Another limitation of the study concerns how adherence was asked of those responsible for categorical questions with yes or no answers that took into account how the child's diet was most of the time (75% of meals). There is no definition of what is the best way to measure adherence to CF methods, this can generate heterogeneity in the way researchers evaluate in their studies.”

It is important that adherence to BLISS in this study is clearly defined so that results so be interpreted in a more meaningful way. Is adherence classed as only those categorised into the ‘all or most’ group, and none adherence is everyone in the 50/50 and fed by parents’ groups? Were participants only offered these 3 response options? What if someone reported that the child self-fed 75% of the time? Do they fall in the ‘all or most’ or 50/50 category and migrated to mixed? There is a need to be completed transparent about the specific questions that were asked regarding the adherence, the response options offered and how the investigators objectively and accurately categorized these responses into groups that meaningfully reflect adherence. Measuring adherence on a continuum or using more clearly defined categories to demonstrate adherence/non-adherence is necessary.

**Answer: Information on how adherence was assessed has been added in the methods section, and the authors are available for further clarification.**

Minor issues

3. The authors need to carefully proof read the document for wording issues and punctuation. For example, there are sometimes full stops both before and after citations in brackets. Page 4 ‘both con consistencies’ which one can assume is meant to say ‘both consistencies’. Page 10, state ‘proposed’ when I think they mean reported? I would recommend changing the terminology from ‘subjected to an intervention’. ‘Subjected’ has different meanings in different contexts but can be inferred as meaning ‘to cause or force someone or something to experience something harmful, unpleasant’.

**Answer: The term 'both con consistencies' has been replaced by the text: "In summary, the mother would prepare meals with some foods in strips or sticks and other foods in mashed or pureed form and allow the child to be spoon-fed and take pieces of food concomitantly at will, without following a regular pattern." The term 'subjected' has been replaced by ‘submitted’ or ‘randomized’, and ‘proposed’ has been replaced by ‘reported’.**

4. Intervention: It needs to clearer whether the intervention was individual or in groups. It can be inferred from page 5, they did it groups of 8 but this is not clear. In that section it reads like there may only be 8 pairs in total in each intervention arm, rather than 8 doing the intervention together in a group.

**Answer: As suggested, the sentence has been changed to:**

“The interventions were conducted in groups of up to eight pairs of mothers and/or caregivers and children, according to the CF randomized method (PLW, BLISS or mixed).”

5. On Page 6 it states that parents could contact the nutritionist, did you measure how many parents did this and how often? It may be pertinent to consider if this was associated with adherence.

**Answer: The authors appreciate your concern. The researchers didn’t consider this possibility of contact as a factor of adherence in this study, as they intend to deepen this topic in another paper. This information was put on the “limitations” section.**

The possibility to contact the nutritionist by phone or email wasn’t evaluated for the adherence to the method in this study.

6. Within materials and methods there is repetition when describing the intervention, on page 4 labelled a/b/c/ then again on page 6 under intervention, please describe this only once.

**Answer: Repeated information has been removed.**

7. Within data collection (page 7) it states ‘food frequency questionnaire and a food record prepared for this study’, please provide further details on the these measures; what specifically they included, how they were developed and their validity/reliability. More information has been added to the section:

**Answer: As informed above, the methods section on adherence to methods have been rewritten and more information has been added.**

“Follow-up data on family adherence to the CF method were collected until the end of the first year of life when children were seven, nine, and 12 months old.

The interview was conducted by a researcher blinded to the method using a standardized questionnaire designed for this study on food consistency and form of offering. It was questioned whether most of the time the food was liquidized, scraped, mashed, sieved, or cut into strips and of the same consistency as the family’s food. Regarding the form of offer, those responsible answered whether the children took the food to their mouths alone or if they received complementary foods by spoon-feeding them up. Answers were provided as yes or no.

At the 7-month interview, by phone, at nine months of age, home visits, and 12 months of age, at the Porto Alegre Clinics Hospital Research Center.

During the COVID-19 pandemic, at 9 months 50.7% (*n*=67) of the sample answered the questionnaires online, and at 12 months 80.3% (*n*=94) answered the online version of the same questionnaires.

Adherence to the method was separated by feeding mode (if children received food from a spoon or took it to their mouths with their hand) and food consistency (if the food was liquidized, scraped, mashed, sieved, or cut into strips and the same consistency of the family’s food). Adherence to PLW was represented by parents who offered food with a spoon (feeding mode) and of a liquidized, scraped, mashed, or sieved consistency (food consistency) most of the time. Adherence to BLISS was represented by children who took food (feeding mode) of the same consistency as their family’s or in strips to their mouths (food consistency), and adherence to the mixed method was represented by those who used alternating feeding modes and consistencies most of the time.”

8. Page 7 – There are some wording issues; ‘The research team called all families to apply food records and ensure that they had no doubts, as well as to ensure the quality of the data.’ What do the authors mean by ‘applying the food records’ do they mean collecting the data? What is meant by ‘ensuring they have no doubts’, do the authors mean that the research team were available to respond to queries about the study or the feeding method should any arise? What did the researchers do if participants did ‘have doubts’? How many participants had 'doubts'? The nutritionists role if participants had 'doubts' needs to clearer as this is mentioned elsewhere in a different way.

**Answer: The section on data collection and compliance has been rewritten and more information was added, to clarify the theme. As written above, the researchers didn’t consider this possibility of contact as a factor of adherence in this study, as they intend to deepen this topic in another paper. This information was put on the “limitations” section.**

The possibility to contact the nutritionist by phone or email wasn’t evaluated for the adherence to the method in this study.

9. Page 7 – Participants were ‘asked some questions regarding food consistency’, what were the questions? Please could the authors clarify if this was the two questions they state or were there more? There are issues with clarity in expression in this sentence that need to be addressed.

**Answer: The section on data collection and compliance has been rewritten and more information added.**

10. Page 7 – states ‘another researcher analysed the answers’, please state how they analysed them. Writing here needs to be more academic, the sentence states they compared the data with the data at 9 months, this could be removed as analysis is covered in the data analysis section, or the specific analyses techniques used to compare the data should be stated here.

**Answer: The section on data collection and compliance has been rewritten and more information added.**

11. Figure 1 a bit misleading with regard to ‘n’. I would recommend reporting the true n – the number of participants who completed the measures at each time-point (with the number missing at each time point noted), not the amount of people eligible for follow up.

**Answer: Figure 1 was redone following the CONSORT recommendations.**

12. Table 2 good be labelled more clearly. I would recommend stating ‘follows mode only’ and ‘follows consistency only’, this makes it clear you are referring to those who follow mode but not consistency, and consistency but not mode, and both.

**Answer: The terms were adjusted according to the reviewer's suggestion.**

13. Page 12 – some issues in interpretation and reporting of adherence findings, please report in a more meaningful way. Do the authors mean by 12 months 96 children were being fed with the mixed method? This does not mean they have ‘adhered’ to that method. It means some have not adhered to their randomised method and migrated to or chosen to follow mixed method. They cannot ‘adhere’ to something they were not allocate to.

**Answer: The way the results are presented in Figures 2 and 3 has been changed.**

“Figures 2 and 3 show the behavior of children regarding feeding method, referred, and food consistency at seven, nine, and twelve months of age. The curves demonstrate that the mixed method was the most followed in consistency and feeding method, followed by the PLW method, and the method that was least followed at all times was the BLISS method.

Regarding the feeding mode (Figure 2), at seven months, 92 (66.2%) children were following the mixed method, 28 (20.1%) were following the PLW method, and 12 (8.6%) were following the BLISS method. At nine months, 89 (64.4%) children were following the mixed method, 34 (24.5%) were following the PLW method, and 9 (6.5%) were following the BLISS method. At 12 months, 96 (69.1%) children were following the mixed method, 21 (15.1%) were following the PLW method, and 18 (12.9%) were following the BLISS method. The mixed method was the most followed by the children, and the BLISS method was followed by the least children at all evaluated ages (seven, nine, and 12 months).

Regarding the consistency of feeding (Figure 3), at seven months 102 (73.4%) children were following the mixed method, 18 (12.9%) were following the PLW method, and 12 (8.6%) were following the BLISS method. At nine months, 84 (60.4%) children were following the mixed method, 16 (11.5%) were following the PLW method, and 32 (23.0%) were following the BLISS method. At 12 months, 133 (95.7%) children were following the mixed method, 2 (1.4%) were following the PLW method, and none were following the BLISS method.”

14. The final paragraph of the discussion is weak and requires further development. There are also typo’s such as ‘..’.

**Answer: The last paragraph of the discussion has been rewritten.**

“This study provides a significant and unprecedented contribution to the field of complementary feeding. As far as we know, it is the first study to evaluate adherence to the major feeding introduction methods described in the scientific literature at three different time points. The study design included personalized nutritional counseling and practical intervention, with the aid of an experimental kitchen equipped for food preparation. However, this study is not exhaustive on the topic of adherence to complementary feeding methods. The authors recommend further studies focusing on standardizing the way adherence is evaluated.”
